# Drug Reaction With Eosinophilia and Systemic Symptoms (DRESS) Following Conversion Surgery for Esophageal Cancer: A Case Report

**DOI:** 10.7759/cureus.89366

**Published:** 2025-08-04

**Authors:** Hiromi Yasuda, Takashi Ichikawa, Shigeyuki Yoshiyama, Masaki Ohi, Yuji Toiyama

**Affiliations:** 1 Department of Gastrointestinal and Pediatric Surgery, Division of Reparative Medicine, Institute of Life Sciences, Graduate School of Medicine, Mie University, Tsu, JPN

**Keywords:** conversion surgery, drug reaction with eosinophilia and systemic symptoms, esophageal cancer, immune checkpoint inhibitor, immune-related adverse event

## Abstract

Conversion surgery is increasingly used for initially unresectable esophageal cancer patients responding to induction therapy. The integration of immune checkpoint inhibitors (ICIs) into standard chemotherapy regimens is expected to increase the number of patients undergoing this approach. However, ICIs can cause immune-related adverse events (irAEs), which are often difficult to diagnose in the postoperative setting. Among these, drug reaction with eosinophilia and systemic symptoms (DRESS) syndrome is a rare but potentially life-threatening complication. A 71-year-old woman with stage IVA esophageal squamous cell carcinoma received induction chemotherapy, including nivolumab, fluorouracil, and cisplatin, followed by successful conversion surgery. Postoperatively, she developed fever, widespread rash, eosinophilia, and liver dysfunction. Initial suspicion was sepsis, and broad-spectrum antibiotics were started. However, repeated tests showed no infection. The retrospective review met the criteria for DRESS syndrome, with the postoperative contrast agent likely triggering the reaction. Supportive care and discontinuation of unnecessary antibiotics led to gradual improvement, and she was discharged for rehabilitation. This case highlights the diagnostic challenge of hypersensitivity reactions such as DRESS in the postoperative setting after ICI-based therapy. Although not a classical irAE, ICI-induced immune dysregulation may have contributed to its development. Awareness of atypical irAEs is essential for timely diagnosis and appropriate management.

## Introduction

Conversion surgery may be considered for patients with locally advanced, initially unresectable esophageal cancer if they show a favorable response to chemotherapy that makes resection feasible. Recently, immune checkpoint inhibitors (ICIs) have been shown to demonstrate efficacy in the treatment of unresectable, advanced esophageal cancer [1,2]. In 2021, the addition of nivolumab to two-drug chemotherapy as first-line therapy and the combination of nivolumab and ipilimumab were proven to be effective in the CheckMate 648 study [2]. Expanded use of ICIs in chemotherapy regimens for esophageal cancer is expected to increase the number of patients eligible for conversion surgery. While ICIs have demonstrated significant efficacy, they are also associated with a variety of immune-related adverse events (irAEs). Among the dermatologic irAEs induced by ICIs, drug reaction with eosinophilia and systemic symptoms (DRESS) syndrome has been reported as extremely rare [3,4]. DRESS is a rare but severe drug-induced hypersensitivity reaction characterized by fever, rash, eosinophilia, and internal organ involvement. The diagnosis is often made using established criteria such as those from the RegiSCAR group, which help differentiate DRESS from other drug eruptions.

Here, we report a case of esophageal cancer in which the patient received ICI-based induction chemotherapy and subsequently developed a clinical course suggestive of DRESS syndrome following conversion surgery. This article was previously presented at the 68th Annual Meeting of the Kansai Thoracic Surgical Association.

## Case presentation

**Figure 1:**
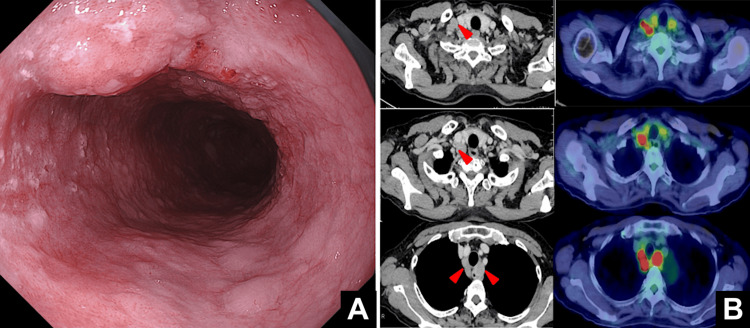
Pre-treatment imaging findings. (A) Pre-treatment endoscopic image showing an elevated lesion in the upper thoracic esophagus. On the anal side of this lesion, a type 0-IIa + IIc lesion extended over approximately four-fifths of the luminal circumference. (B) Pre-treatment CT (left) and PET-CT (right) images showing the right supraclavicular lymph node, the 106recR lymph node, and the 106recR/106recL lymph node enlargement with FDG uptake, indicating lymph node metastasis. The enlarged lymph nodes are indicated by red arrowheads on the images.

A 71-year-old woman with a medical history of right renal and bladder cancer was being monitored for renal cancer when mediastinal lymph node swelling was incidentally detected. Further evaluation confirmed the diagnosis of thoracic esophageal cancer. Upper gastrointestinal endoscopy revealed an elevated lesion with an adjacent type 0-IIc area, extending over approximately four-fifths of the luminal circumference (Figure 1A).

Computed tomography (CT) demonstrated enlargement of several lymph nodes, including the recurrent laryngeal nerve and right supraclavicular nodes. Positron emission tomography-computed tomography (PET-CT) showed 18F-fluorodeoxyglucose (FDG) uptake in these nodes, indicating lymph node metastasis (Figure 1B). The metastatic lymph node closely abutted the membranous portion of the trachea, raising suspicion of invasion. Based on these findings, the patient was diagnosed with squamous cell carcinoma of the upper to middle thoracic esophagus (Ut-Mt), cT4 (trachea), cN2, cM1a, and cStage IVA according to the 12th edition of the Japanese Classification of Esophageal Cancer.

**Figure 2:**
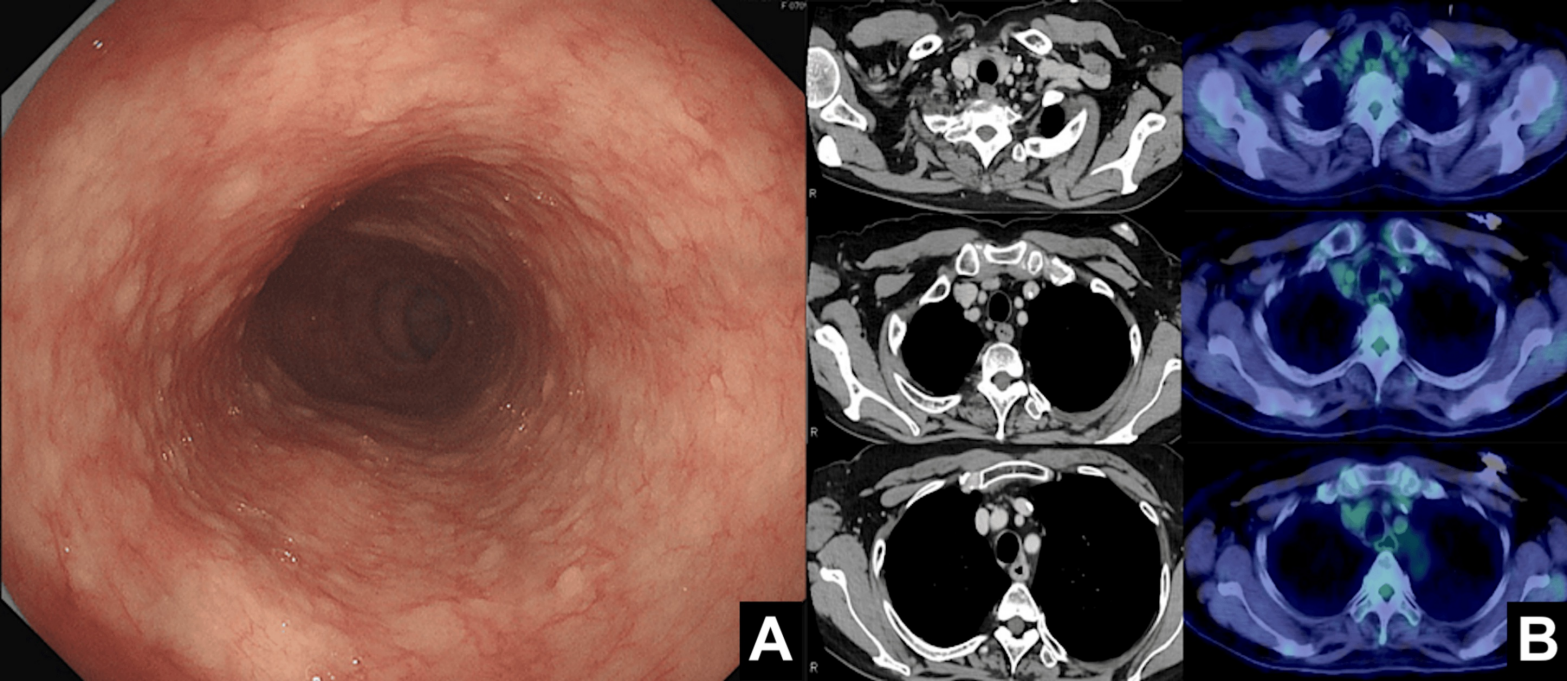
Post-treatment imaging findings. (A) Endoscopic image after chemotherapy showing a reduction in the size of the protruded lesion, indicating a favorable response to induction therapy. (B) Post-treatment CT (left) and PET-CT (right) images showing resolution of the lymph node enlargement and FDG uptake in the same areas, suggesting a favorable response to induction chemotherapy.

Diagnosed with unresectable advanced esophageal cancer, the patient was started on a chemotherapy regimen of 5-fluorouracil and cisplatin (FP) plus nivolumab. The protocol included 5-fluorouracil (800 mg/m²/day) administered intravenously on days one to five every three weeks and cisplatin (80 mg/m²) administered intravenously. After six cycles of FP plus nivolumab followed by an additional cycle of nivolumab monotherapy, imaging studies showed a favorable response: the protruded lesion was reduced on upper gastrointestinal endoscopy (Figure 2A), lymph node shrinkage was observed on CT, and PET-CT revealed no residual uptake in the lymph nodes (Figure 2B). Consequently, curative resection became feasible, and conversion surgery was planned.

The conversion surgery was performed 25 days after the final chemotherapy cycle. The procedure, robot-assisted subtotal esophagectomy with three-field lymph node dissection and cervical esophagogastric anastomosis, was completed successfully. On postoperative day (POD) four, a routine contrast-enhanced CT scan triggered an anaphylactic shock attributed to the contrast medium, which was managed with steroids. Although the patient remained stable, an observed increase in inflammatory markers raised concern for the possibility of a minor anastomotic leak. As a precaution, fasting was initiated, and antibiotic therapy with tazobactam/piperacillin was started.

**Figure 3:**
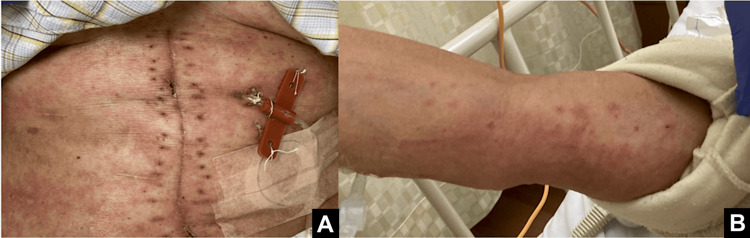
Skin rash on the trunk and upper limbs. A skin rash was observed on the (A) trunk and (B) upper limbs. Photographs showing a widespread erythematous rash that developed on the trunk and upper extremities following postoperative antibiotic administration.

Three days after antibiotic initiation, a skin rash developed on the trunk and limbs (Figure 3). One of the four blood cultures was positive for Gram-positive cocci, prompting the removal of the central venous catheter and the initiation of teicoplanin. Despite these measures, the fever persisted. On the fourth day of teicoplanin treatment, the patient developed a temperature of 40°C and hypotension, resulting in shock.

**Figure 4:**
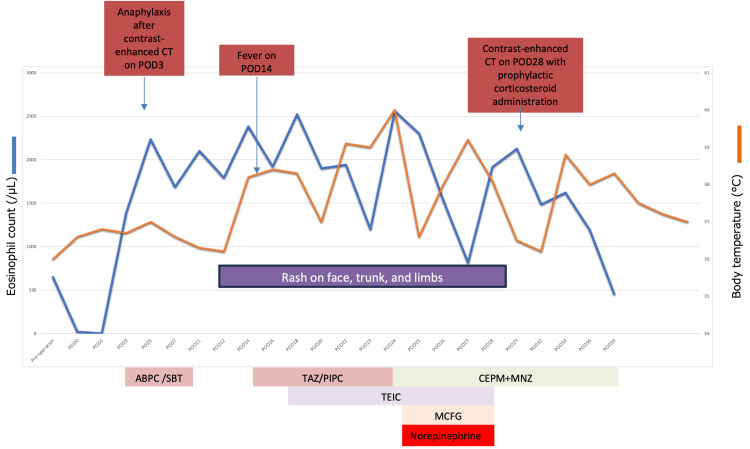
Clinical course following the conversion surgery. Clinical course following the conversion surgery. The timeline graph illustrates the postoperative clinical parameters from the preoperative period to POD 39. The upper panel depicts the changes in body temperature (max: 40℃) and eosinophil count (peak: 2560/µL). The lower panel lists the antibiotics administered during the same period. The periods for norepinephrine administration and the presence of a skin rash are also indicated. The arrows highlight the following key events: the day of anaphylaxis after contrast-enhanced CT, the onset of the initial fever, and the day when contrast-enhanced CT was performed again with prophylactic corticosteroid administration. ABPC/SBT: ampicillin/sulbactam; TAZ/PIPC: tazobactam/piperacillin; CEPM: cefepime; MNZ: metronidazole; TEIC: teicoplanin; MCFG: micafungin; POD: postoperative day

Although the source of the infection remained unidentified, the shock was assumed to be caused by sepsis. Vasopressor support with norepinephrine was initiated along with continued antibiotic therapy. Blood tests revealed leukocytosis, eosinophilia, elevated transaminases, and a high procalcitonin level. Although the high procalcitonin suggested infection, imaging and further microbiological evaluations, such as repeat blood cultures, pleural fluid cultures, and magnetic resonance imaging for spondylitis and cholangitis, were all negative. On POD 28, a contrast-enhanced CT scan was performed following prophylactic corticosteroid administration (methylprednisolone 40 mg IV at one, seven, and 13 hours prior) to identify the source of fever. Subsequently, the eosinophil count decreased, the fever resolved, and the rash improved (Figure 4).

Based on this clinical course, DRESS syndrome was suspected. The antibiotics were tapered and discontinued, resulting in no recurrence of eosinophilia, normalization of liver function, and stabilization of the overall condition. Human herpesvirus 6 (HHV-6) reactivation testing was negative. Following clinical improvement, the patient was transferred to a rehabilitation facility on POD 60.

## Discussion

DRESS syndrome is a rare but severe hypersensitivity reaction that typically occurs two to eight weeks after the administration of certain culprit drugs, such as antiepileptic agents and medications for hyperuricemia. It is characterized by fever, skin rash, eosinophilia, lymphadenopathy, and internal organ involvement [5]. However, DRESS is not widely recognized among general clinicians, particularly in the postoperative setting, which can lead to delays in diagnosis and appropriate treatment.

Dysfunction of regulatory T cells (Tregs) is believed to play a central role in the pathogenesis of severe drug eruptions such as DRESS syndrome. Recently, ICIs have also been reported as potential triggers of severe drug eruptions, including DRESS syndrome, by disrupting immune tolerance and promoting hypersensitivity reactions to concomitantly administered medications [6]. Furthermore, emerging case reports have documented occurrences of DRESS syndrome following ICI therapy in other cancer types, such as melanoma, renal cell carcinoma, and gastric cancer, supporting the potential causal relationship between ICIs and DRESS development [3,7,8].

This case highlights the diagnostic challenges posed by atypical irAEs following ICI-based therapy and conversion surgery for esophageal cancer. While ICIs have significantly improved outcomes in unresectable esophageal cancer and expanded the indications for conversion surgery [1,2], they are also associated with a wide range of irAEs, including rare but severe cutaneous manifestations such as DRESS syndrome.

Distinguishing between postoperative infections and irAEs, such as DRESS, remains difficult due to overlapping clinical features, including fever, rash, and circulatory instability [9]. In particular, elevated serum procalcitonin (PCT) levels have been reported in patients with DRESS even in the absence of bacterial infection, which can mimic sepsis and complicate the diagnostic process [10].

In our case, the initial presentation was suspected to be sepsis. Despite broad-spectrum antibiotic therapy, no source of the infection was identified. Retrospective analysis revealed early eosinophilia and a maculopapular rash, indicating a non-infectious etiology.

The patient met six of the seven Japanese Research Committee on Severe Cutaneous Adverse Reaction (J-SCAR) criteria for DRESS, including (1) prolonged symptoms; (2) fever ≥38°C; (3) delayed macular rash; (4) hematologic abnormalities; (5) lymphadenopathy; and (6) liver dysfunction. HHV-6 reactivation was negative. However, corticosteroid premedication for contrast imaging and withdrawal of antibiotics led to symptom improvement, further supporting the diagnosis [11].

It is important to note that the timing of HHV-6 testing is critical; if sampling is delayed, reactivation may be missed. Thus, cases with characteristic clinical features but negative HHV-6 results may still be classified as "atypical DRESS." This is consistent with a report by Ingen-Housz-Oro et al., which showed HHV-6 reactivation in only 38.5% of ICI-associated DRESS cases [3].

Although corticosteroids are typically used over a prolonged period with tapering, our patient received only three prophylactic doses of methylprednisolone for contrast allergy and no maintenance immunosuppression. Nevertheless, all symptoms resolved without relapse, suggesting a mild form of DRESS or spontaneous resolution following discontinuation of the suspected agent.

To rule out other irAEs, we conducted a thorough evaluation. Thyroid and adrenal function were normal, and imaging showed no evidence of pneumonitis or colitis.

To our knowledge, this is the first reported case of suspected DRESS syndrome following conversion surgery in an esophageal cancer patient treated with ICI therapy. Although causality cannot be definitively proven, the clinical course and temporal association strongly suggest a link.

## Conclusions

This case illustrates the diagnostic challenges of immune-related hypersensitivity reactions in the postoperative period, especially in patients treated with ICIs. Awareness of conditions such as DRESS syndrome is crucial for timely diagnosis and appropriate management. When postoperative fever occurs with eosinophilia and no identifiable infection, clinicians should consider non-infectious causes, including DRESS, and consider corticosteroid therapy. As the use of ICIs increases, systematic evaluation and careful monitoring of postoperative patients are essential to improve outcomes and prevent delays in diagnosing immune-related adverse events.
